# Three-dimensional quantification of mandibular asymmetries in Caucasian adult patients with different sagittal and vertical skeletal patterns. A cone beam study using 3D segmentation and mirroring procedures

**DOI:** 10.1186/s13005-023-00400-2

**Published:** 2023-12-14

**Authors:** Pilar España-Pamplona, Natalia Zamora-Martinez, Beatriz Tarazona-Álvarez, Valmy Pangrazio-Kulbersh, Vanessa Paredes-Gallardo

**Affiliations:** 1 Faculty of Medicine and Dentistry, School of Dentistry, University of Valencia, C/ Gasco Oliag, 1, Valencia, 46010 Spain; 2 Department of Orthodontics, School of Dentistry, University of Detroit Mercy, Detroit, MI USA

**Keywords:** Mandibular asymmetry, Skeletal patterns, 3D segmentation, CBCT

## Abstract

**Introduction:**

An accurate identification of mandibular asymmetries is required by modern orthodontics and orthognathic surgery to improve diagnosis and treatment planning of such deformities. Although craniofacial deformities are very frequent pathologies, some types of asymmetries can be very difficult to assess without the proper diagnostic tools. The purpose of this study was to implement the usage of three-dimensional (3D) segmentation procedures to identify asymmetries at the mandibular level in adult patients with different vertical and sagittal patterns where the asymmetries could go unnoticed at the observational level.

**Methods:**

The study sample comprised 60 adult patients (33 women and 27 men, aged between 18 and 60 years). Subjects were divided into 3 sagittal and vertical skeletal groups. CBCT images were segmented, mirrored and voxel-based registered with reference landmarks using ITK-SNAP® and 3DSlicer® software’s. 3D surface models were constructed to evaluate the degree of asymmetry at different anatomical levels.

**Results:**

There was a degree of asymmetry, with the left hemimandible tending to contain the right one (0.123 ± 0.270 mm (CI95% 0.036–0.222; *p* < 0.001). Although the subjects under study did not present significant differences between mandibular asymmetries and their sagittal or vertical skeletal pattern (*p* = 0.809 and *p* = 0.453, respectively), a statistically significant difference has been found depending on the anatomical region (*p* < 0.001; CI95%=1.020–1.021), being higher in the condyle, followed by the ramus and the corpus.

**Conclusions:**

Although mandibular asymmetries cannot be correlated with vertical and sagittal skeletal patterns in symmetric patients, knowledge about 3D segmentation procedures and color maps can provide valuable information to identify mandibular asymmetries.

## Introduction

The prevalence of facial asymmetry ranges from 8.7 to 23.3% of the general population [1]. Facial asymmetries often affect the lower third of the face, which can be explained by the long period of time involved in mandibular growth [2, 3]. Several authors associate the presence of mandibular asymmetry with skeletal discrepancies, due to its relationship with transverse [4], sagittal [3, 5–8] or vertical alterations [7], suggesting that facial asymmetries are more common among patients presenting crossbite, skeletal class II or III malocclusions, and/or dolichocephalic patterns [3, 5, 7–10].

Regarding the affected side, Severt y Proffit [3] demonstrated that when the chin was deviated, there was an 80% of chance that the deviation would shift to the left. On the contrary, other authors [2, 3, 9] showed a tendency towards the right side in regions like the condyle or the temporary area. As for the anatomical region authors such as You et al. [11], found that both the condyle and the jaw body contributed equally to the presence of mandibular asymmetries.

Facial symmetry can be defined by the equal position of two points on each side of the face in relation to the mid-sagittal plane [2] and the relation of the central regions such as menton, lips and the subnasal region with this mid-sagittal plane. Although most faces may appear well balanced and symmetrical in clinical observation [1], radiographic analyses indicate the presence of asymmetry as a common feature of all faces. In the past, treatment planning and assessment of mandibular asymmetries was limited to 2D diagnostic radiographs [12, 13] where the accuracy provided was not sufficient [8, 12–14]. The introduction of 3D segmentation techniques [15–19] with CBCT [20–24] allowed to create virtual models where a more detailed analysis of the position of an asymmetry [7, 15] can be performed and opens interesting new possibilities. By delineating the shape of the structures [16, 17, 25] using quantitative and qualitative morphological information [18, 19], these techniques have become of increasing interest to the medical image analysis.

The hypothesis of our study was that the patient’s sagittal and vertical skeletal patterns may be determining factors for presenting mandibular asymmetry. The main objectives of the present study were to find out, with the aid of a previously validated 3D segmentation technique, whether the presence of mandibular asymmetries was related to the skeletal malocclusion and to assess whether the anatomical region could contribute to its severity. The purpose of this study was to implement the usage of three-dimensional (3D) segmentation procedures to identify asymmetries at the mandibular level in adult patients with different vertical and sagittal patterns where the asymmetries could go unnoticed at the observational level.

## Materials and methods

### Sample

The study sample was 60 adult Caucasian patients seeking treatment at the Dental Clinic of the master’s Program in Orthodontics at the University of Valencia (Spain) (between January 2015 and November 2019) The University of Valencia Ethics Committee for Research Involving Human Subjects (Ref: H1488134666059) approved the study. The research followed the Declaration of Helsinki principles and STROBE guidelines, and all patients provided their informed consent.

The initial sample size consisted of 132 patients, of which 72 were excluded after application of the inclusion and exclusion criteria. The final sample consisted of 60 patients, 33 women (55%) and 27 men (45%). Mean age was 31.7 ± 10.6 years ranging between 18 and 60 years.

The sample size was determined by a pilot study were power analysis, indicated that data from at least 60 participants would yield a confidence level of 95% and provide an 80% probability of detecting a large effect size (f = 0.4) associated to the interaction term, at a confidence level of 95%, where the variable used was the deviation of the menton.

The inclusion criteria consisted of participants presenting permanent dentition and less than 2 mm of deviation of the menton when were clinically analysed. According to the studies [3, 18], we can consider an asymmetry as clinically relevant when there is more than 2 mm of deviation of the menton. All the participants had a complete cranial CBCT with a field of view that included Basion, Glabela, Porion and Menton structures as part of their initial records.

The exclusion criteria were (1) presence of missing, included teeth and/or ectopic eruptions (2), presence of large metal restorations that could provoke interference in 3D assessment of CBCTs (3), previous orthodontic treatment (4), presence of dental or facial traumatisms and (5) craniofacial anomalies or syndromes.

The participants were classified according to the sagittal skeletal pattern using the ANB angle described by Steiner [26] (class I, ANB: 2º±2º, class II, ANB: > 4º and class III, ANS < 0º) and the vertical skeletal pattern using the Frankfort mandibular plane angle (FMA) described by Ricketts [27] (mesocephalic = 22º-28º; brachycephalic < 22º; dolichocephalic > 28º).

**Fig. 1:**
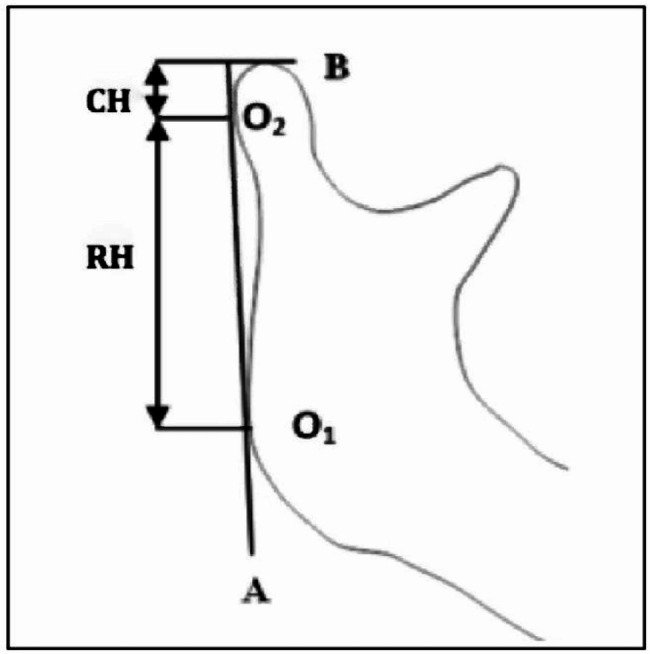
Mandibular regions delimited according to Habets et al. [28]technique

The mandibles were delimited according to Habets et al. [28] technique. Three regions were considered (condyle, ramus, and corpus) (Fig. 1).

### Equipment

All the scans were performed with the same CBCT machine (Dental Picasso Master 3D®, EWOO technology, Republic of Korea, 2005) with a standard FOV of 200 × 150 and a scanning time of 15 s.

3D virtual models were constructed with a reconstruction time of 1 min 5 s. and the scanning angle covering 360º and capturing 592 slices. The voxel size used was 0.4 × 0.4 × 0.4 mm. The tube voltage range was 40–90 kV with an intensity range of 2–10 mA. Focal size was 0.5 mm and base size was 180 × 170 cm. The data files generated were of 450 megabytes each.

The CBCTs were taken by placing the patients in the natural head position (NHP), according to Park et al. [29]. Three reference planes were established to ensure that the patient position was properly oriented: the axial plane, defined as the occlusal plane, the coronal plane: perpendicular to the axial plane, at the height of the mesiobuccal cusp of the maxillary first molars and the sagittal plane, perpendicular to both planes, crossing through the midpoint between the medial edges of the orbits.

#### Semi-automatic segmentation and mirroring

**Fig. 2:**
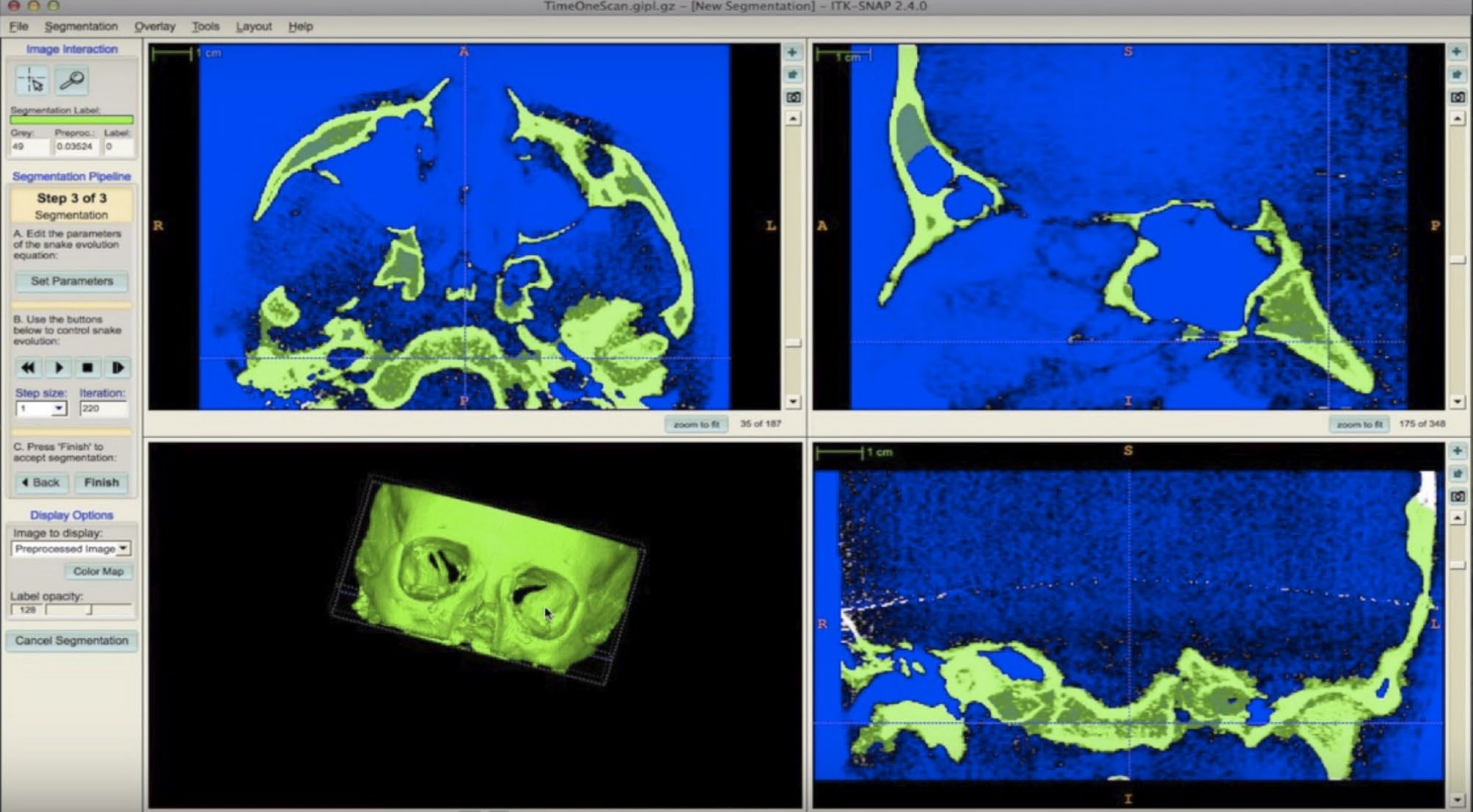
3D semi-automatic segmentation process with ITK-SNAP® software (open source software, http://www.itksnap.org)

**Fig. 3:**
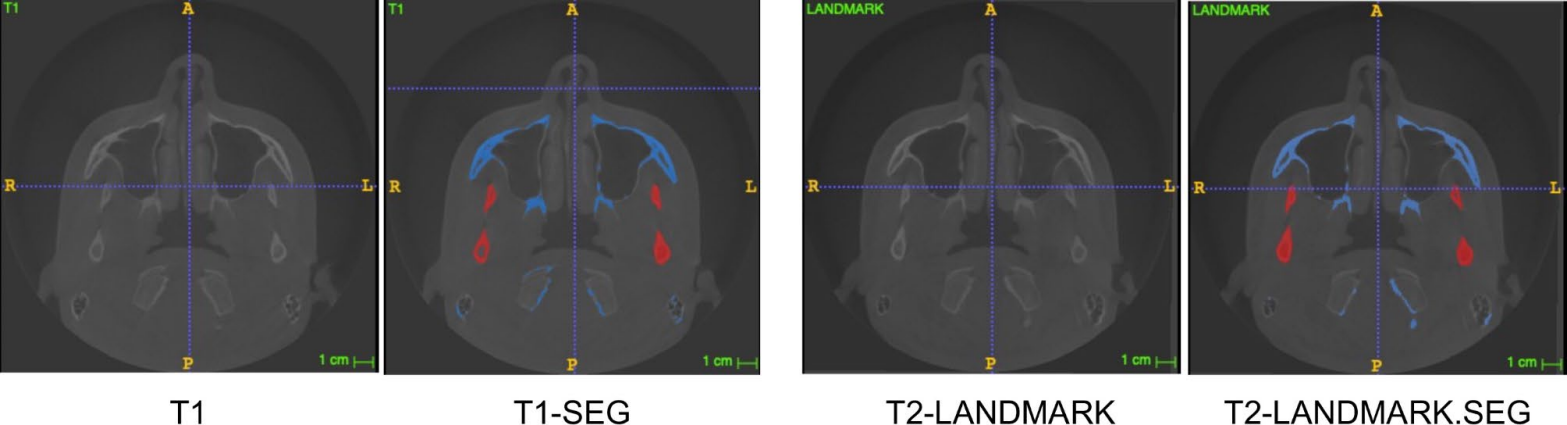
Semi-automatic segmentation and mirroring process with ITK-SNAP® software (open source software, http://www.itksnap.org)

To carry out the segmentation of the jaws and the volumetric reconstruction, a validated model previously published was used [17–19, 25, 30, 31]. The original DICOM files (T1) stored with Dolphin® 11.5 software were converted to “.nrrd” files (T1.nrrd) using ITK-SNAP® software. After selecting the region of interest (ROI) visible in the frontal, sagittal and transverse sections and adding bubbles in the area to be segmented, the software automatically segmented the bone tissue (T1-SEG.nrrd) (Fig. 2). Using the “Reorient image” tool, a mirror image of the original file was obtained (T2-Landmark.nrrd) and segmented (T2-LandmarkSEG.nrrd). With this procedure two images of each patient were stored (Fig. 3).

#### Cranial base registration

**Fig. 4:**
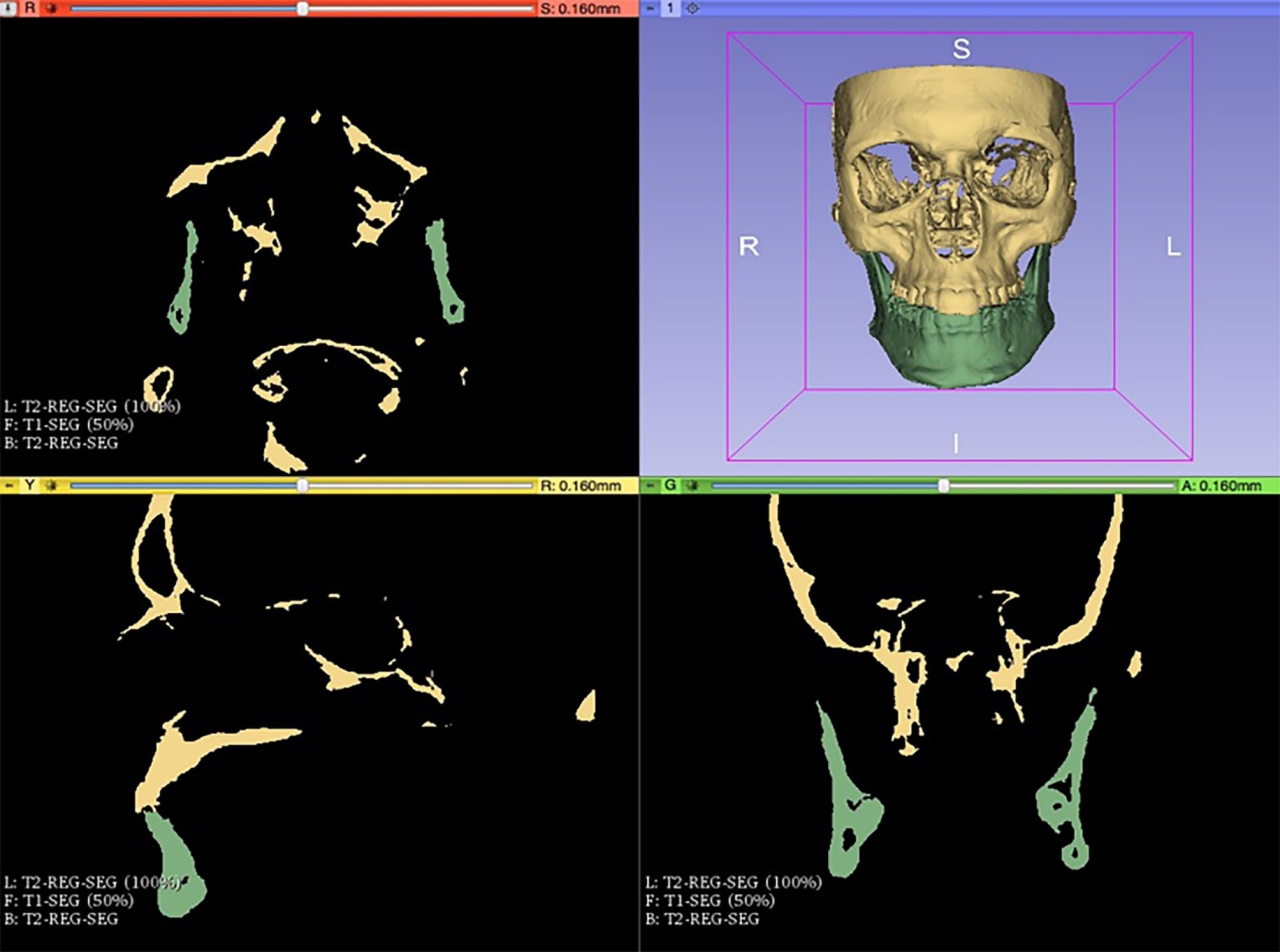
Cranial base registration of the 3D model with 3D Slicer® software (www.slicer.org). In yellow the cranial base and the upper jaw, and in green, the lower jaw

A cranial base register of the 3D models was performed (Fig. 4). It is important since it allows to assess mandibular asymmetry in relation to the craniofacial complex, differentiating true mandibular asymmetries from those derived from the position of the glenoid fossa. Using the 3D Slicer® software’s “Landmark registration” tool, stable reference points were located on the anterior cranial base (Nasion, Basion and Anterior Nasal Spine) and maxillae, eliminating pitch, roll and yaw errors. Both the original image (T2-reg-seg.nrrd) and its mirror image (T2-reg-scan.nrrd) were then registered according to these reference points.

#### Creation of surface models

**Fig. 5:**
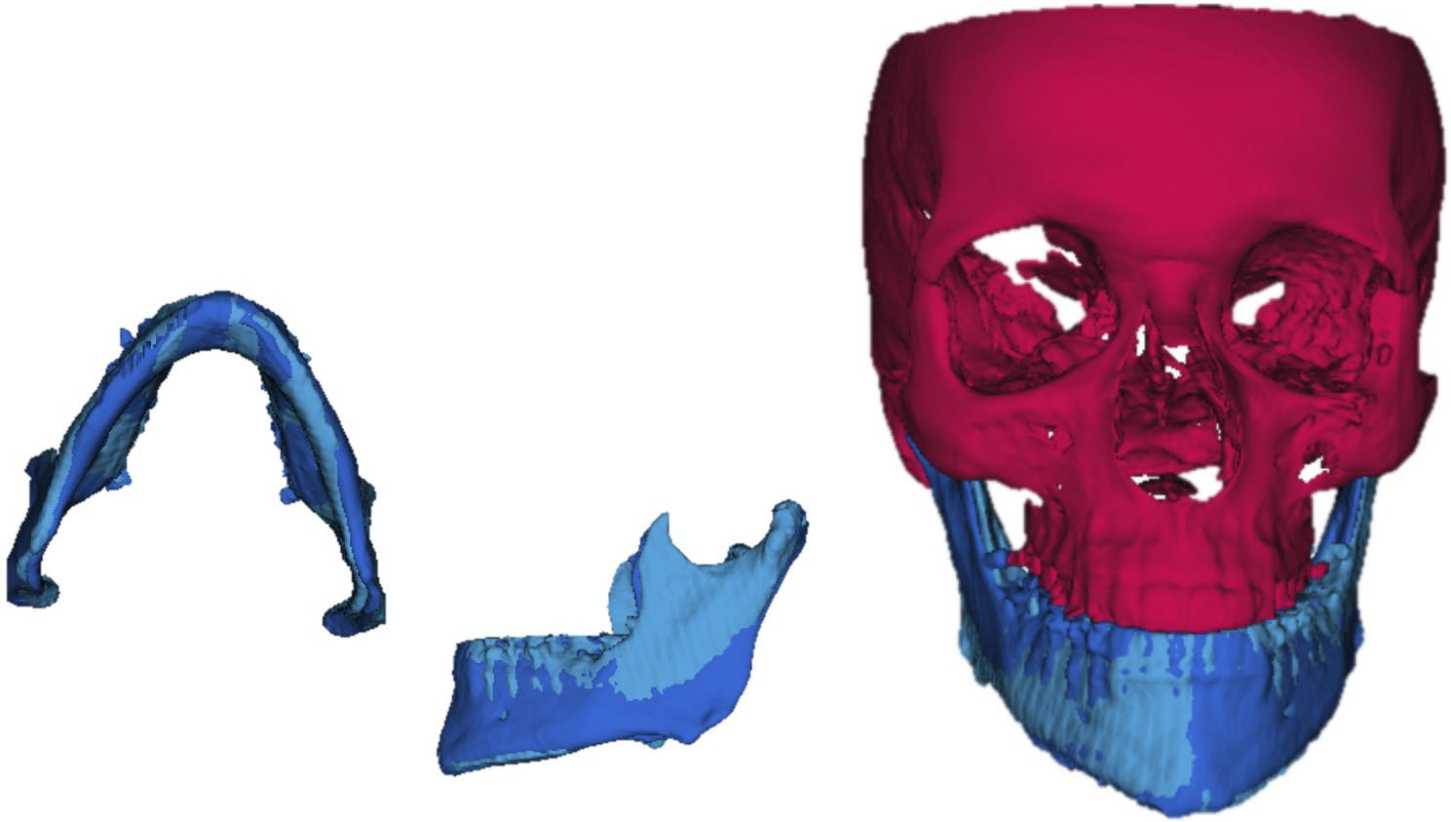
Superimposed surface models with 3D Slicer® software (www.slicer.org). In red, the cranial base, in dark blue the mandible of the original CBCT and in light blue, the mandible of the mirror image

Using 3D Slicer® software, a voxel-based registration superimposition of the original CBCT and the mirror image was performed. Once the surface models had been properly superimposed, the corresponding jaws could be isolated (Fig. 5). Since thanks to the registration on the cranial base, the positional asymmetries have been eliminated, superimposing the original CBCT mandibular model and their mirror image, the morphological asymmetries can be determined.

#### Creation of hemimandibles

**Fig. 6:**
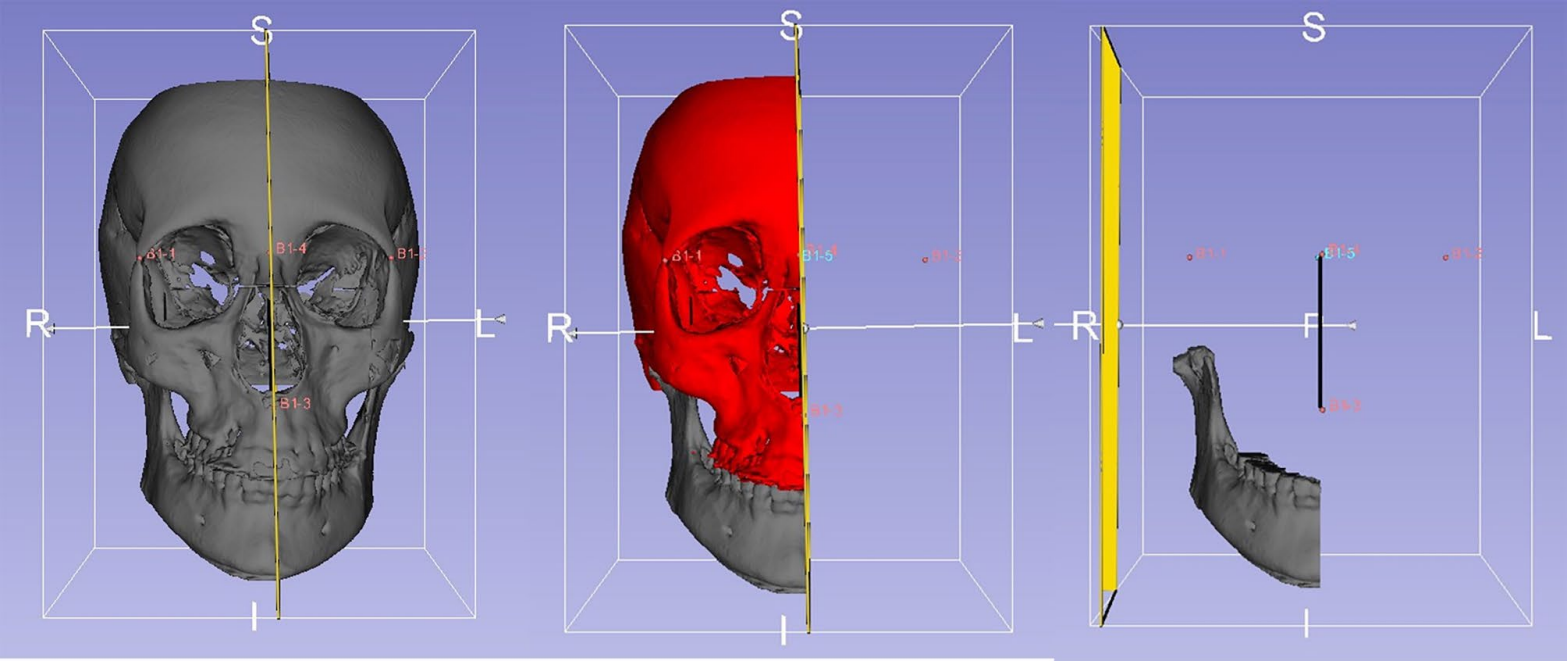
Frontal view of the surface model with the midsagittal plane, frontal view of the surface model of the right side and frontal view of the hemimandible model of the right side correctly registered on the cranial base. 3D Slicer® software (www.slicer.org)

To be able to split each mandible in two equal sides, the mid-sagittal plane was located. The midsagittal plane was defined as that passing through the Anterior Nasal Spine, Nasion, and the midpoint between the innermost points of the frontozygomatic suture, at the outer margin of the right and left orbital rims (points used in Ricketts and Grummons frontal cephalometry [32]) for being stable and easily identifiable. With “Q3DC”, “Angle Planes” and “Easy Clip” tools from 3D Slicer® software and taking the right side as a reference, hemimandible models were created (Fig. 6).

#### Color maps and measurement registration

**Fig. 7:**
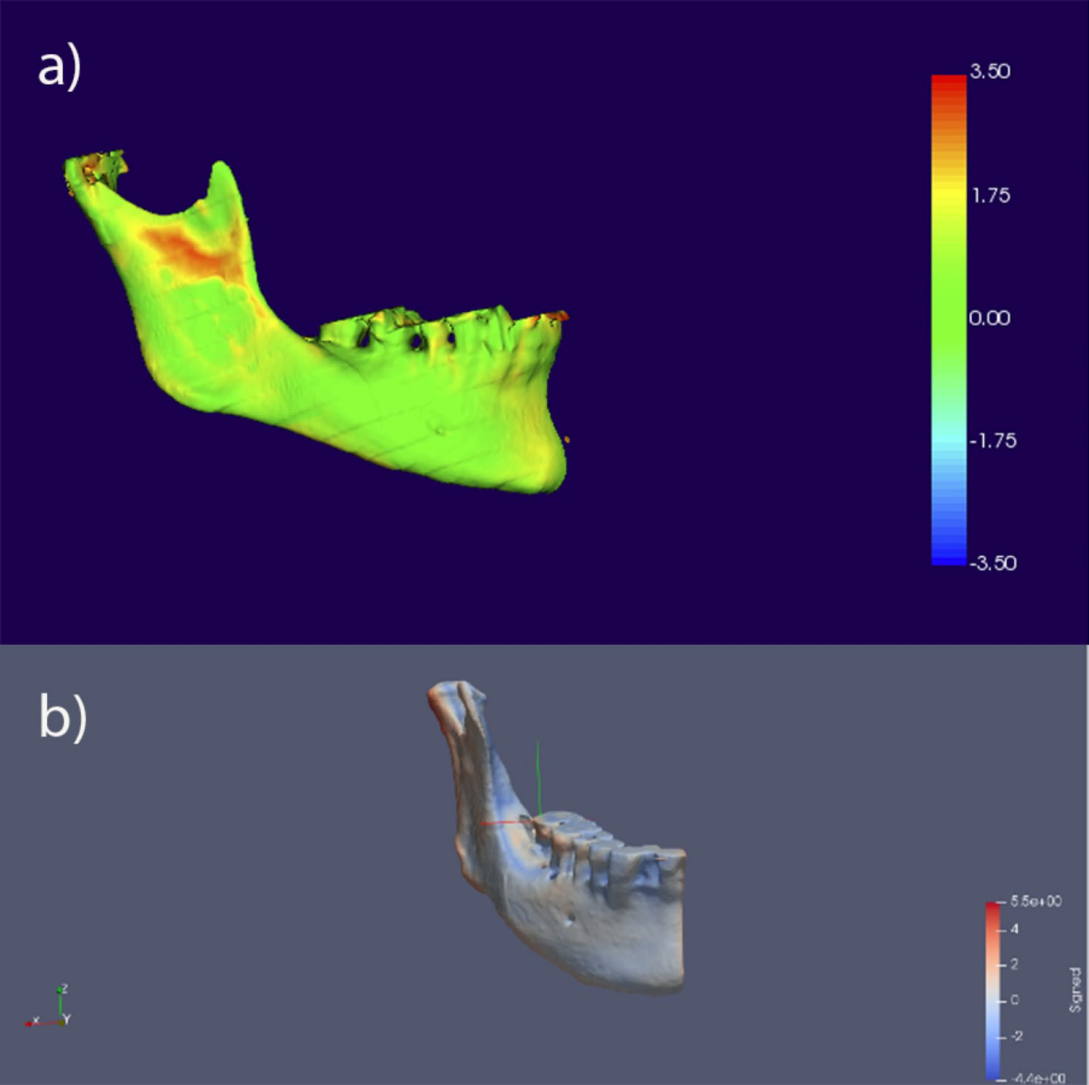
Example of a color map representing the positive and negative distances of superimposed hemimandibles with Paraview® software

Mandibular asymmetries were quantified using color maps (Fig. 7). They quantify the degree of changes between the two models by applying different colors on their surfaces. Each color represents a different level of variation, indicating not only the distance of the asymmetry but also the direction of the measurement. The asymmetry was assessed as the difference between an hemimandible and the mirror image of the contralateral hemimandible. In order to simplify the process, measurements were made only by taking the right hemimandible as a reference. To register the asymmetry in millimeters (mm), SPHARM-PDM software and Procrustes analysis were used [18] to compute correspondent point-based models of all the hemimandibular surfaces per each patient. Each single point on the right hemimandible (reference hemimandible) was considered as the origin of a vector that connected to the equivalent point on the left hemimandible. A range of ± 2 mm was established as relevant because the sample had < 2 mm of deviation of the Menton when the participants were clinically analysed. The distances between equivalent points of one and another hemimandible were measured and a color scale was set. Asymmetries were evaluated using two different concepts [1], signed distance and [2] absolute distance. The signed distance assessed the directional measurement of the asymmetry, providing complete information of which hemimandible tended to contain the other (by a positive (+) or a negative sign (-)). Thus, each measurement had a positive sign (+) when the left hemimandible tended to contain the right whereas a negative sign (-) occurred when the right hemimandible tended to contain the left. It also corresponded to a vector of three components (axis X, Y, Z). Absolute distance assessed the module of the signed distance, quantifying the amount of asymmetry regardless of its direction. For a better understanding, the process can be explained with an example: i.e., if in half of the points the asymmetry was + 2 mm with the left hemimandible containing the right mandible and in the other half was − 2 mm, with the right hemimandible containing the left one, the average distance would be 0; but undoubtedly, the asymmetry exists and that´s why the signed distance needed to be considered.

After undergoing a two-month training period using five sample CBCTs, the main researcher (P.E), now skilled and calibrated, took measurements from each of the 60 CBCTs, making a total of 10,505,915 measurements. To estimate measurement error, and assess intra- and inter-observer correlation and reproducibility, a second subset of measurements of 15 subjects were taken by the same researcher (P.E) and by a second researcher (B.T), following the same methods and parameters as described above.

### Statistical analysis

The data obtained was entered in an Excel® datasheet (Microsoft Office® for Mac 2011 package). Statistical analyses of data distribution and significance were performed using SPSS® software (version 15.0; IBM Corp., Armonk, NY, USA).

To assess whether the asymmetry depended on independent factors such as sagittal or vertical skeletal pattern or anatomical region, a general linear one-way ANOVA model was estimated. Bonferroni´s test was used as a multiple comparison test between different categories.

The Kolmogorov-Smirnov test was used because the subgroups of patients always consist of more than 15 cases. The homogeneity of variances was ensured by the Levene test. The level of significance used was 5% (α = 0.05).

A general linear ANOVA model reached a power of 77.6% to detect a large effect size in the difference in mean distance between groups of individuals (class I, II, III or brachycephalic, mesocephalic or dolichocephalic), at a 95% confidence level. In order to evaluate differences in mandibular asymmetry according to the anatomical region, power analysis showed that mean distances of 0.85, 1.0 and 1.15 mm ± 0.33 mm would provide a maximum significant effect (f = 0.4). The same ANOVA model of point-based measurements (n = 10,505,915) achieved maximum power (> 99.9%) under the same conditions.

## Results

The ICC (intra-class correlation coefficient) estimated had a value of over 0.89, indicating that method error was low and that there was high intra-operator reliability. At the same time, the ICC for inter-operator reliability was 0.76, suggesting high repeatability of the measurements.

According to the sagittal skeletal pattern the sample was distributed as follows: 21 Class I (35%), 20 Class II (33.3%) and 19 Class III subjects (31,6%).

Regarding the vertical skeletal pattern, the sample was divided in 22 brachycephalic (36,6%), 19 mesocephalic (31,6%), and 19 dolichocephalic subjects (31,6%).

In the point-based model, several thousand points per patient were recorded to assess the response variables such as the distance vectors and components. Specifically, an average of 175,098 points per subject were registered, accumulating a total sample of 10,505,915 points.

When assessing the global degree of the asymmetry through the point-based model analysis, before the participants were divided in groups, the mean signed distance found was 0.123 ± 0.270 mm (CI95%=0.036–0.222; *p* < 0.001, t-test) (Table 1). The degree of asymmetry evaluated was positive and significant, with the left hemimandible tending to contain the right hemimandible.

**Table 1:** Analysis of the signed distance (mm) and absolute distance (mm) according to the sagittal and skeletal pattern

	SIGNED DISTANCE (mm)							ABSOLUTE DISTANCE (mm)						
	N (patients)	Mean ± s.d	Minimun	Maximun	Median	CI95%	*P* value*	N (patients)	Mean ± s.d	Minimun	Maximun	Median	CI95%	*P* value*
**TOTAL**	60	0.123±0.270	-0.343	1.664	0.076	0.036–0.222	*p* < 0.005**		1.012±0.270	0.545	2.035	0.849	0.937–1.090	*p* < 0.001**
**Sagittal Skeletal Pattern**														
Class I	21	0.121±0.307	-0.332	0.864	0.122		*p* = 0.809	21	0.979±0.244	0.545	1.647	0.995		*p* = 0.977
Class II	20	0.092±0.356	-0.343	0.925	0.066			20	1.001±0.225	0.645	1.374	0.893		
Class III	19	0.130±0.296	-0.224	1.664	0.087			19	0.983±0.225	0.884	2.035	0.934		
**Vertical Skeletal Pattern**														
Mesocephalic	19	0.0169±0.337	-0.744	1.664	0.087		*p* = 0.453	19	1.021±0.622	0.678	2.034	1.065		*p* = 0.671
Brachycefalic	22	0.066±0.421	-0.354	0.955	0.001			22	1.080±0.321	0.595	2.035	0.889		
Dolichocefalic	19	0.123±0.447	-0.343	0.845	0.088			19	0.936±0.303	0.545	1.559	0.994		

*p value, F test, ** statistically significant, s.d = standard deviation, CI = Confidence interval

In the analysis of the absolute distance, a mean of 1.012 ± 0.270 mm (CI95%=0.937–1.090; *p* < 0.001, t-test) was obtained, indicating also a significant degree of asymmetry.

### Asymmetry and sagittal skeletal pattern

Class III skeletal individuals had a mean signed distance (mean 0.130 ± 0.269 mm) greater than class I (0.121 ± 0.307 mm) or class II individuals (0.092 ± 0.356 mm), but without being statistically significant (*p* = 0.809, F test) (Table 1).

**Fig. 8:**
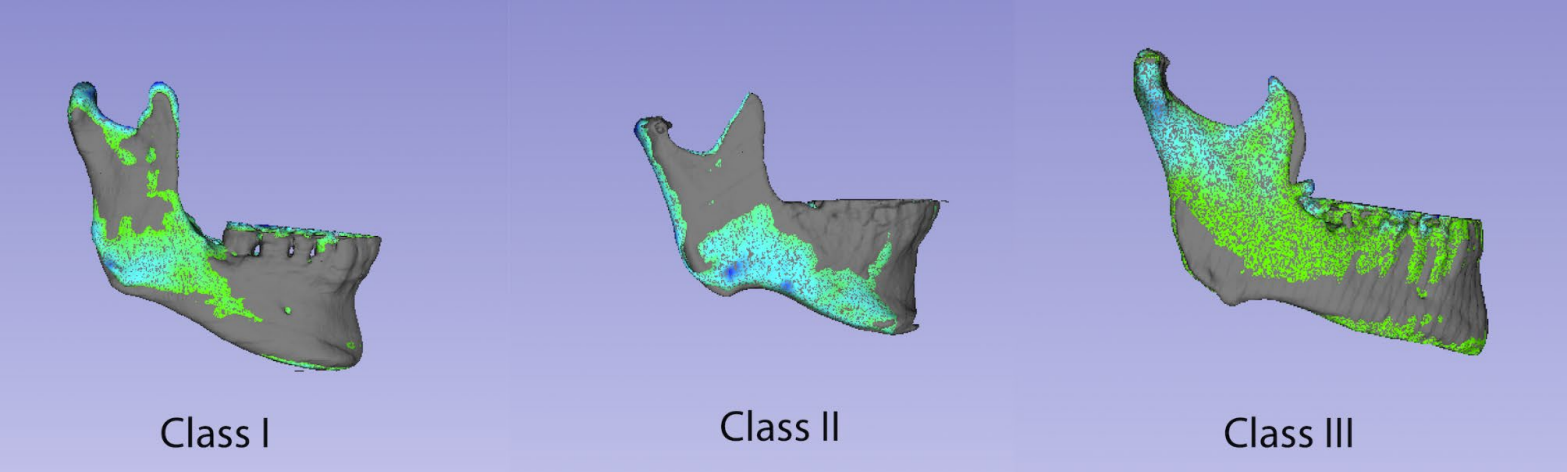
Examples of color map assessment of hemimandibles with different sagittal skeletal patterns. Color scale representing the positive and negative distances of superimposed hemimandibles (dark blue: -2 mm; blue: -1 mm; grey: no difference; green: 0 mm; yellow: +1 mm; red: +2 mm)

In relation to the absolute distance, the mean differences between classes were around 1 mm (class I = 0.979 ± 0.244 mm; class II = 1.001 ± 0.255 mm; class III = 0.983 ± 0.225 mm) and no statistically significant results were found (*p* = 0.977, F test). Thus, the degree of the asymmetry did not depend on the skeletal class. An example of the color map representing the positive and negative distances of superimposed hemimandibles in different sagittal skeletal patterns is shown in Fig. 8.

### Asymmetry and vertical skeletal pattern

The signed distance revealed no statistical differences (*p* = 0.453, F test) in the mean between mesocephalic (0.169 ± 0.337 mm) brachychepalic (0.066 ± 0.421 mm) and dolichocephalic individuals (0.123 ± 0.447 mm) (Table 1). In relation to the absolute distances, the mean differences between patterns were around 1 mm (mesocephalic = 1.021 ± 0.622 mm; brachycephalic = 1.080 ± 0.321 mm; dolichocephalic = 0.936 ± 0.303 mm), with no statistically significant results (*p* = 0.671, F test). Here, again, the degree of the asymmetry does not depend on the vertical skeletal pattern.

**Fig. 9:**
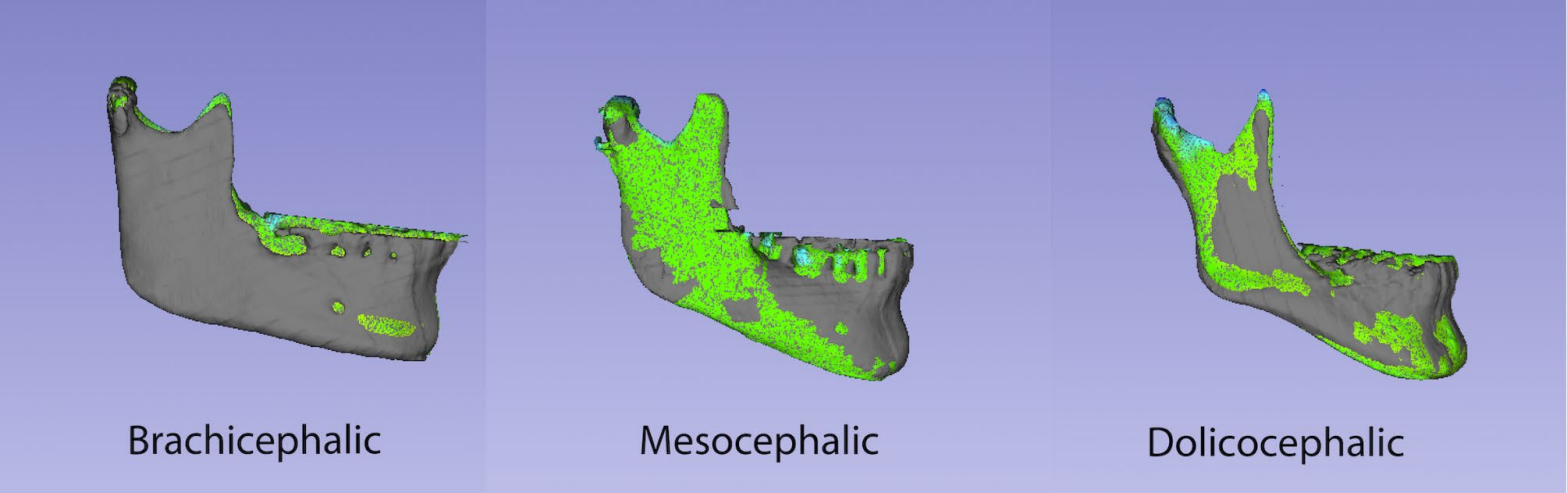
Examples of color map assessment of hemimandibles with different vertical skeletal patterns. Color scale representing the positive and negative distances of superimposed hemimandibles (dark blue: -2 mm; blue: -1 mm; grey: no difference; green: 0 mm; yellow: +1 mm; red: +2 mm)

In Fig. 9 differences of the three vertical skeletal patterns are represented in a color map.

### Asymmetry and anatomical region

Asymmetries were assessed through the point-based model analysis (Table 2). All points of each patient were classified according to the anatomical region to which they belonged. The overall distribution of point was as follows: condyle = 602,560 points (5.8%), ramus = 2,380,325 points (22.7%), body = 7,523,030 points (71.5%). Overall, the average signed distance was 0.139 ± 1.413 mm. The greater asymmetry was found at the condyle (mean = 0.723 ± 1.567 mm), followed by the ramus (0.256 ± 1.470 mm) and the corpus (0.051 ± 1.234 mm). The degree of asymmetry was statistically significant in the three anatomical regions, with a greater difference in the condyle (*p* < 0.001; CI95%=1,040–1 042).

**Table 2:** Analysis of the signed distance (mm) and absolute distance (mm) according to the anatomical region

Anatomical region	SIGNED DISTANCE (mm)							ABSOLUTE DISTANCE (mm)						
	N (landmarks) /%	Mean ±s.d	Minimun	Maximun	Median	CI 95%	*p* value*	N (landmarks)/%	Mean±s.d	Minimun	Maximun	Median	CI 95%	*p* value*
Total	10,505,915/ (100)%	0.139± 1.413	-8.002	20.002	0.009	1.040–1.042		10,505,915/ (100)%	1.003± 0.752	0.000	20.002	0.675	1.020–1.021	
Condyle	602,560/ (5.8%)	0.723± 1.567	-3.443	8.990	0.477		*p* < 0.001**	602,560/ (5.8%)	1.283± 1.221	0.000	8.990	1.111		*p* < 0.001**
Ramus	2,380,325/ (22.7%)	0.256± 1.470	-4.584		0.088		*p* < 0.001**	2,380,325/ (22.7%)	1.137± 1.040	0.000	20.002	0.878		*p* < 0.001**
Corpus	7,523,030/ (71.5%)	0.051± 1.234	-8.002	8.999	0.003		*p* < 0.001**	7,523,030/ (71.5%)	0.993± 0.981	0.000	8.999	0.699		*p* < 0.001**

*p value, t test ** statistically significant s.d = standart deviation

The average absolute distance was 1.003 ± 0.752 mm, with the mandibular condyle having a greater asymmetry (mean = 1.283 ± 1.221 mm) than the ramus (1.137 ± 1.040 mm) or the corpus (0.993 ± 0. 981 mm). The difference here was statistically significant (*p* < 0.001, t-test; CI95%=1.020–1.021), indicating that the asymmetry was greater in the condyle.

## Discussion

The 3D segmentation protocol used in the present study followed the guidelines promoted by other authors [16–19, 25, 31]. One of the advantages of quantifying mandibular asymmetries with 3D segmentation procedures, CBCT and color maps is that both sides of the mandible can be precisely and easily compared in terms of size and structure, leaving behind the errors produced by the incorrect choice of reference points in linear measurements or by anatomical variations that may complicate the proper detection on 3D structures and contours [10, 16, 33]. In the present study, asymmetry was defined as the difference between an hemimandible and the mirror image of the contralateral one. The most difficult step in the segmentation protocol was the creation of the hemimandibles, choosing the midsagittal plane as we know that the accuracy of landmarks in 3D is not easy. Recently, comparisons using bilateral indexes [34] or mirror images [35] with CBCT have been published. Although our results showed that the left hemimandible tended to contain the right one, as other authors did [3], it is difficult to define which side should be considered as the altered one. To simplify the process, measurements were made only by taking the right hemimandible as the reference. According to Al-Hadidi et al. [18] no statistically significant differences were found when the asymmetries were quantified taking as reference the individual´s right or left side, which demonstrates the consistency of our method.

In our study, CBCT scans were introduced in two software packages (ITK-Snap® and 3DSlicer®) to create a fully automated superimposition and to obtain segmented hemimandibles. A voxel-based registration method on the cranial base was also performed in order to eliminate pitch, roll and yaw errors (movements of the jaw seen from the frontal plane equal to what the head would do when saying yes, maybe and no, respectively). The main advantage is that there is a direct calculation when only grey values ​​are used, so that the accuracy is not limited by segmentation errors as in surface-based methods [24, 30]. This is important because it allows to evaluate the symmetry of the mandible with respect to the rest of the craniofacial complex, differentiating true mandibular asymmetries from those derived from differences in the position of the glenoid fossa [16, 33].

The images of our study were oriented in the natural head position so as not to mask or accentuate the asymmetries [29]. Although some studies that investigated the influence of the position of the head before the acquisition of the CBCT on the accuracy of the 3D measurements concluded that measurements based on 3D CBCT surface images were accurate and small variations in the position of the patient’s head did not influence the accuracy of the measurements [37], other authors [29, 30] affirmed that the number of directional changes in each plane of space was strongly influenced by the orientation of the head.

The degree of asymmetry found in the present study through the analysis of the signed distance, that is, the directional measurement of the asymmetry, was positive and significant, but it is difficult to justify an average distance of 0.139 mm as being clinically relevant. The same happens with the analysis of the absolute distance, which is the quantification of the amount of asymmetry regardless of its direction. One of the studies [9] that analyzed the same parameters, also found less than 1 mm of asymmetry in all patients of their study, concluding and not considering it as being clinically relevant. It should be noted that no consensus has been established in the literature as what is considered asymmetry and what is not. Some authors [3, 18, 25] considered that mandibular asymmetry is present when there is more than 2 mm of deviation of the chin but others [36] considered it only when the chin presents a deviation of 4 mm or more. In general, for all analyses of point-based models with large sample sizes, it is very easy to find statistically significant differences, even though these are well below levels of clinical relevance.

In our work, the analysis of the signed distance and the absolute distance found that there was no statistically significant relationship between mandibular asymmetries and sagittal or vertical skeletal patterns. Only Class III patients had a positive but no significant asymmetry when compared to Class I and Class II subjects as in other studies [3, 7]. Some authors [8] agree with our results, not supporting the idea of a statistically significant relationship between skeletal discrepancy and mandibular asymmetry. However, some other authors have suggested that a significant relationship exists between individual´s sagittal or vertical skeletal pattern and the presence of mandibular asymmetries [7, 25, 38]. It is possible that the discrepancies between studies could be due to the sample size used in each single case [11, 25].

Regarding the point-based model measurements of the signed and the absolute distance of the three anatomical regions analyzed, statistically significant differences were found between them, been greater in the condyle. Our results were in line to others reported in the literature [7, 10, 11, 15, 39], where the condyle tended to be the most asymmetrical region. The mandibular asymmetry is a result of a complex compensation of the morphology of different regions, such as condyles, ramus and mandibular body. In many cases, one region could mask the asymmetry of other one, keeping a symmetric mandibular position related to cranial base, especially in symmetric patients with less than 2 mm of the deviation of the menton.

## Conclusions

The results of our study rejected the hypothesis that the patient’s sagittal and vertical skeletal patterns may be determining factors for presenting mandibular asymmetry in clinically symmetric patients. Although no statistically relevant differences were encountered, we did find a statistically significant difference between both hemimandibles, with the left tending to contain the right one. The statistically relevant asymmetry also found at the condylar region corroborates the complexity of the structure. Knowledge about 3D segmentation procedures and color maps provide valuable information for orthodontic and surgical interventions associated with mandibular asymmetries.

The results could be not significant because of a few intrinsic and unpredictable variables related to the cranial growth with its adaptation and compensation capabilities, but the absence of significance does not mean that there cannot be any relationship between skeletal features and asymmetries, so further studies are necessary.

## Data Availability

We would be ready to provide further information about our data and methods if you desire it.
